# Impacts of online and group perinatal education: a mixed methods study protocol for the optimization of perinatal health services

**DOI:** 10.1186/s12913-018-3204-9

**Published:** 2018-05-29

**Authors:** Geneviève Roch, Roxane Borgès Da Silva, Francine de Montigny, Holly O. Witteman, Tamarha Pierce, Sonia Semenic, Julie Poissant, André-Anne Parent, Deena White, Nils Chaillet, Carl-Ardy Dubois, Mathieu Ouimet, Geneviève Lapointe, Stéphane Turcotte, Alexandre Prud’homme, Geneviève Painchaud Guérard, Marie-Pierre Gagnon

**Affiliations:** 1 Faculty of Nursing, Université Laval, 1050 avenue de la Médecine, Québec, QC G1V 0A6 Canada; 2 CHU de Québec Research Centre – Université Laval, Hôpital Saint-François d’Assise, 10 rue de l’Espinay, Québec, QC G1L 3L5 Canada; 3 Centre intégré de santé et de services sociaux de Chaudière-Appalaches, Hôtel-Dieu de Lévis, 143 rue Wolfe, Lévis, QC G6V 3Z1 Canada; 4 Université de Montréal Public Health Research Institute, 7101 avenue du Parc, Montréal, QC H3N 1X9 Canada; 5 Faculty of Nursing, Université de Montréal, 2375, chemin de la Côte-Ste-Catherine, Montréal, QC H3T 1A8 Canada; 6 Department of Nursing, Université du Québec en Outaouais, 283 boulevard Alexandre-Taché CP 1250, Gatineau, QC J8X 3X7 Canada; 7 Faculty of Medicine, Université Laval, 1050 avenue de la Médecine, Québec City, QC G1V 0A6 Canada; 8 School of Psychology, Université Laval, 2325 Allée des Bibliothèques, Québec City, QC G1V 0A6 Canada; 9 Ingram School of Nursing, McGill University, 680 Sherbrooke West, Montréal, QC H3A 2M7 Canada; 10 Institut national de santé publique du Québec, 945 av Wolfe, Québec City, QC G1V 5B3 Canada; 11 School of Social Work, Université de Montréal, 3150 rue Jean-Brillant, Montréal, QC H3T 1N8 Canada; 12 Département de sociologie, Université de Montréal, 3150 rue Jean-Brillant, Montréal, QC H3T 1N8 Canada; 13 Department of Political Science, Faculty of Social Sciences, Université Laval, 1030 avenue des Sciences Humaines, Québec, QC G1V 0A6 Canada; 14 School of Public Health, Université de Montréal, 7101 avenue du Parc, Montréal, QC H3N 1X9 Canada

**Keywords:** Prenatal education, Perinatal care, Pregnancy, Childbirth education, Online education, Community health networks, Community health services, Health status indicators, Mixed methods

## Abstract

**Background:**

Prenatal education is a core component of perinatal care and services provided by health institutions. Whereas group prenatal education is the most common educational model, some health institutions have opted to implement online prenatal education to address accessibility issues as well as the evolving needs of future parents. Various studies have shown that prenatal education can be effective in acquisition of knowledge on labour and delivery, reducing psychological distress and maximising father’s involvement. However, these results may depend on educational material, organization, format and content. Furthermore, the effectiveness of online prenatal education compared to group prenatal education remains unclear in the literature. This project aims to evaluate the impacts of group prenatal education and online prenatal education on health determinants and users’ health status, as well as on networks of perinatal educational services maintained with community-based partners.

**Methods:**

This multipronged mixed methods study uses a collaborative research approach to integrate and mobilize knowledge throughout the process. It consists of: 1) a prospective cohort study with quantitative data collection and qualitative interviews with future and new parents; and 2) a multiple case study integrating documentary sources and interviews with stakeholders involved in the implementation of perinatal information service networks and collaborations with community partners. Perinatal health indicators and determinants will be compared between prenatal education groups (group prenatal education and online prenatal education) and standard care without these prenatal education services (control group).

**Discussion:**

This study will provide knowledge about the impact of online prenatal education as a new technological service delivery model compared to traditional group prenatal education. Indicators related to the complementarity of these interventions and those available in community settings will refine our understanding of regional perinatal services networks. Results will assist decision-making regarding service organization and delivery models of prenatal education services.

**Protocol version:**

Version 1 (February 9 2018).

## Background

Prenatal information is a decisive determinant of health choices made by pregnant women and their partners as they move through the continuum of perinatal services [1, 2]. Considering the myriad of information sources publicly available and their variable quality [3–7], prenatal education remains a health promotion strategy at the core of perinatal care and services provided by health and social services centers [8–10] [S-10] and is supported by public policies [10, 11]. Group prenatal education is one of the most common educational models [12]. Various studies have shown that group prenatal education can be effective in the preparation for labour and delivery, reducing anxiety and maximising partners’ involvement. However, these results depend on the organization, format, and content of the educational services [13–17]. In order to address accessibility issues as well as the evolving needs of future parents, some health and social services centers have opted to recommend or implement online prenatal education, while still offering group prenatal education. Decision makers, however, are concerned about the impacts of this new educational mode on the efficacy of health services networks. In a restructuring context where the deployment of online education opens the door to new complementary prenatal education to group education, it is important to understand the contribution of these two educational modes on health determinants and users’ perinatal health [18]. Because of the heterogeneity of delivery modes [9], evidence of prenatal education effectiveness and impact is scarce or contradictory for group prenatal education [12, 14–17, 19] and very limited for online prenatal education [20–22], although online education may address the needs of certain users and improve accessibility [23–29]. Within a health promotion context, prenatal education delivered by health and social services centers could be improved by being integrated into a continuum of perinatal information in partnership with existing community services networks [30]. Several studies show that networking may contribute to health system effectiveness, but structural characteristics and collaborations with community partners surrounding prenatal education and information remain unknown [31–33]. There is thus an urgent need to collect robust data on the impacts of group prenatal education and online prenatal education, and to consolidate perinatal information networks with community partners.

The aim of this project is to evaluate the impacts of group prenatal education and online prenatal education provided or recommended by health and social services centers on health determinants and users’ health status, as well as on networks of perinatal educational services maintained with community-based partners. Specific objectives are to: 1) document the characteristics of group prenatal education and online prenatal education and contribute to their optimization; 2) evaluate the impacts of group prenatal education and online prenatal education on health determinants and the perinatal health status of parents; 3) evaluate characteristics and collaborations related to perinatal educational services within which group prenatal education and online prenatal education are offered, with community-based partners.

## Methods

This multipronged study uses convergent mixed methods through a collaborative research approach to integrate and mobilize knowledge [34, 35]. More precisely, it will consist of 1) a prospective cohort study with quantitative data collection and qualitative interviews with future and new parents and 2) a multiple case study integrating documentary sources and interviews with stakeholders involved in the implementation of perinatal information service networks and collaborations with community partners. The complementarity of the quantitative and qualitative data will provide a broader perspective on perinatal information sources and networks in order to evaluate the impacts of prenatal education.

### Participating sites

The study will be conducted within the geographic territories covered by two health and social services centers located in adjacent regions in the province of Québec, Canada, with an approximate total area of 34,000 km^2^ and total population of 1,162,000 inhabitants. Created in 2015, these regional institutions are responsible for the provision of health care and services within their territories and for binding agreements with partner organizations (e.g. community organizations, medical clinics, network clinics, etc.) [36]. The health and social services centers are providing similar group prenatal education, with some variations related to their resources and specific population needs. Both are currently using an online prenatal education interface developed by a private provider [37]. These institutions also have access to a perinatal information source developed by the Ministry of Health and Social Services of Québec to maintain the harmonization of content [38].

### Participants

For the cohort study, women will be eligible if they: a) are at the beginning of their pregnancy (10 to 20 weeks); b) live within the targeted geographic territories; c) are fluent in French; d) have not given birth previously and e) have a valid email address and access to internet. Male and female partners of women meeting these criteria will be eligible as partners. Partners who already had children with another woman will also be eligible. For qualitative interviews, parents will be eligible if they: a) have a 6- to 12-week-old infant; b) attended group prenatal education or online prenatal education recommended by participant sites; c) live within the targeted geographic territories; d) are fluent in French. For the multiple case study, prenatal education stakeholders (managers and health professionals) will be eligible if they: a) are working within the participating sites or related services networks; b) are interested in sharing their understanding of structural characteristics and determinants of collaboration between health and social services centers and community partners involved in group prenatal education and online prenatal education offer; and c) have been in their position for at least 3 months. These stakeholders will be identified with the help of collaborators from participating sites.

### Outcomes

Primary and secondary outcomes related to perinatal health and perinatal health determinants were identified from a literature review on group prenatal education effects [18, 39]. Based on studies that demonstrated significant effects of group prenatal education, the main outcome for health determinant is perinatal knowledge [13, 40] and will be measured with an adapted version of the Health Pregnancies Knowledge Survey [41]. The questionnaire will be adapted in collaboration with prenatal education trainers from the different participant training sites, in order to ensure that all knowledge items are covered in group and online prenatal education.

The main secondary outcome is psychological distress, measured with a validated French version of the 12-item General Health Questionnaire [42] and considered as the most important outcome for perinatal health measures (i.e., main outcome measuring a health determinant). Other secondary outcomes include: breastfeeding self-efficacy, assessed with a French version of the Breastfeeding Self-Efficacy Scale Short-Form [43, 44]; anxiety, assessed with a validated French version of the State-Trait Anxiety Inventory [45]; self-efficacy in the parenting role, assessed with a French version of the Parent Expectations Survey [46, 47]; depression, assessed with a validated French version of the Edinburgh Postnatal Depression Scale [48, 49]; concern about labour and birth, assessed with a French version of the Oxford Worries about Labour Scale [50]; control during childbirth, assessed with a French version of the Labour Agentry Scale [51]; personal control in pain relief during childbirth, assessed with a French version of the Personal Control in Pain Relief Scale [52], breastfeeding status and birth weight. A back translation process [53, 54] will be used to translate English versions of the Oxford Worries about Labour Scale, Labour Agentry Scale and Personal Control in Pain Relief Scale to French. Data on sociodemographic characteristics, pregnancy and childbirth history, and prenatal information sources consulted during pregnancy will also be collected as potential confounding factors. All questionnaires will be pre-tested with a test-retest procedure in order to assess their reliability [55].

### Data collection

#### Administrative data collection

Throughout the entire duration of the project, administrative data needed to establish a general portrait of prenatal education use will be collected and updated with health and social services centers managers. These data will include different characteristics of the organization, format and content such as number and duration of group prenatal education meetings, health professionals involved in group prenatal education, themes covered in group prenatal education and online prenatal education, mode and fees for accessing group prenatal education and online prenatal education, and sources used for the development of group prenatal education and online prenatal education. Administrative data will also be obtained from the online prenatal education provider and will include access data, registration data and users’ satisfaction data.

#### Time measurements (cohort study)

Table 1 presents the distribution of outcomes measurements through time for the cohort study. Time measurements are calculated according to the continuum of services of participating institutions. The first questionnaire (T1) will be completed between the 10th and 20th week of pregnancy, in order to reach participants before the prenatal education period. The second questionnaire (T2) will be sent at 33 weeks of pregnancy, in order to reach participants after the prenatal education period. The third and last questionnaire (T3) will be sent 6 weeks after the expected date of birth. All questionnaires will be sent by email and completed online.

**Table 1:** Distribution of outcomes measures through time

	(T1) 10–20 weeks of pregnancy		(T2) 33 weeks of pregnancy		(T3) 6 weeks after child birth	
	Pregnant women	Partners	Pregnant women	Partners	Mothers	Partners
Main outcome measure						
Health Pregnancies Knowledge Survey	✓	✓	✓	✓		
Secondary outcomes measures						
General Health Questionnaire	✓	✓	✓	✓	✓	✓
Breastfeeding Self-Efficacy Scale	✓	✓	✓	✓	✓	✓
State-Trait Anxiety Inventory	✓	✓	✓	✓	✓	✓
Parent Expectations Survey	✓	✓	✓	✓	✓	✓
Edinburgh Postnatal Depression Scale	✓	✓	✓	✓	✓	✓
Sociodemographics characteristics	✓	✓				
Pregnancy history			✓			
Prenatal information sources			✓	✓		
Childbirth history					✓	
Breastfeeding status					✓	
Oxford Worries about Labour Scale					✓	✓
Labour Agentry Scale					✓	✓
Birth weight					✓	✓

*✓*Outcome measured

#### Qualitative interviews

Semi-structured individual qualitative interviews with parents will be based on an interview guide developed according to the Interactive Quality Health Education Outcomes Model [56]. Interviews will be conducted by phone in order to facilitate participation. Each interview will last approximately 45 min. Mothers and partners from each participating site and each prenatal education mode (group or online prenatal education) will be recruited according to a stratified sampling until data saturation is reached (expected *N* = 40) [57–59]. Qualitative interviews will be held simultaneously with the cohort study.

#### Network data collection

**Fig. 1:**
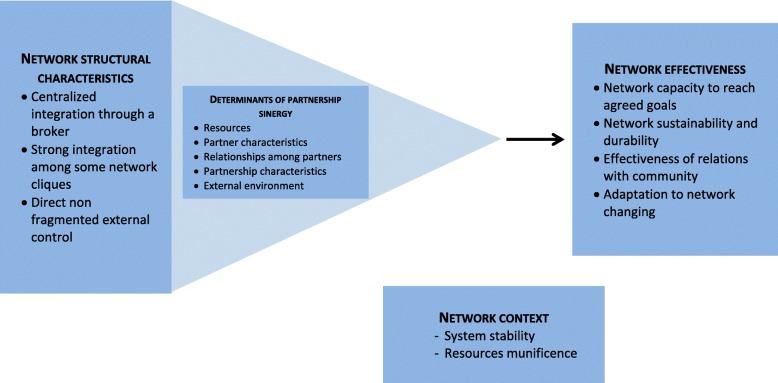
Networks and partnerships effectiveness reference framework. Legend: Adapted from Lasker et al. (2001) and Turrini et al. (2010)

In order to evaluate structural characteristics of efficient networks and collaborations, individual qualitative interviews with prenatal education stakeholders will be held in the two participating health and social services centers and their related community-based organizations (expected *N* = 45). The interview guide will be developed from a reference framework inspired by the work of Turrini et al. [31] for efficient network characteristics, and Lasker et al. [60] for partnerships functioning (Fig. 1). An adaptation of the Social Network Analysis Tool [61] will also be used in order to estimate how these characteristics and determinants may consolidate group prenatal education, online prenatal education and perinatal information. Each interview will last approximately 45 min. These interviews will be held simultaneously with the cohort study and will be completed by documentary sources provided by the participating sites.

#### Recruitment strategies

For the cohort study, all questionnaires will be completed in electronic format and data kept on a secured server hosted by the principal investigator’s institution. Pregnant women and their partners will be recruited at their first contact point with participating sites, namely at their first ultrasound test or prenatal meeting. In ultrasound clinics, a bookmark providing connection information will be given by receptionists when parents attend the first dating ultrasound. A research assistant will then be responsible to meet potential participants in the waiting room, provide them with the necessary information and give them the opportunity to complete the consent form and start answering the questionnaire on an iPad. Potential participants will also be free to keep the bookmark and complete the questionnaire later at home. In prenatal clinics, the bookmark and project information will be given by nurses to future parents, which will then be free to complete the online consent form and questionnaire at home. Posters will also be displayed in all participating sites, with the possibility for potential participants to contact the principal investigator or project coordinator directly if they want to participate. Once participants are registered, follow-up will be done by email or phone.

For qualitative interviews, parents will be recruited at their first postnatal clinic encounter in participating health and social services centers (e.g. immunization clinics, breastfeeding clinics, etc.). A bookmark with the research team coordinates and project information will be given by nurses to new parents, who will be invited to contact the research team in order to verify their eligibility and participate to the project.

For the multiple case study, expert stakeholders will be recruited through a snowball sampling technique starting with the health and social services centers’ decision-makers initially involved in the study.

#### Incentives and retention strategies

In order to prevent loss to follow-up during the cohort study, automatic email reminders will be sent twice after the sending of T1, T2 and T3 questionnaires. If the questionnaire is not completed after that, the research coordinator or a research assistant will call the participant as a last reminder or to record the reason for abandonment. All participants will be eligible for the drawing of six iPads, with chances to win proportionately increasing with the number of completed questionnaires (one to three).

### Statistical analysis

#### Sample size

Assuming an effect size of 0.36 (for perinatal knowledge with or without prenatal education) and a 1:1:2 allocation between groups (group prenatal education: online prenatal education: without prenatal education) [13], a power of 80% and a bilateral test threshold of 0.025, a total of 445 pregnant women and 445 partners (2 groups of 111 with prenatal education and 1 group of 223 without prenatal education) is required at the third measurement time. An ongoing longitudinal study conducted by our research team with new parents in the Québec region allows us to expect a participation rate of 80% for partners and a retention rate of 70% at the end of the three measuring times. An initial sample size of 795 pregnant women and up to 795 partners is therefore anticipated. A second power calculation based on Jakubiec et al. data [40] was done for the most important secondary outcome (psychological distress) and resulted in a smaller sample size. Births by territory data suggest a sufficient pool to recruit the required sample size within 3 to 4 months.

#### Quantitative analysis

Descriptive analysis will be conducted at the three time points. For the main outcome (measured twice), difference between prenatal education groups (group prenatal education and online prenatal education) and standard care without these prenatal education services will be calculated. Bivariate linear regression models will then be used to measure the association between this difference and secondary outcomes. Bivariate linear regression models will be used to compare prenatal education groups (group prenatal education and online prenatal education) to the absence of these prenatal education services for health determinants measured twice. Non-multicollinearity, normality of residuals and homogeneity of variances will be verified and a variable transformation will be performed if these postulates are not met. Outcomes measured at the three time points will be analyzed with bivariate repeated measures models. For continuous and categorical outcomes, mixed models and generalized estimating equation models will be used respectively. For the generalized estimating equation models, binomial distribution will be used for binary outcomes and multinomial distribution for outcomes with multiple categories. Multiple imputation will be used for randomly distributed missing data. Depending on the results, sensitivity analysis may be performed for geographic regions, health establishments providing prenatal education, group or online prenatal education format, exposure level to online prenatal education and healthcare providers involved in pregnancy follow-up. All statistical models will be adjusted for sociodemographic data, use of other information sources and pregnancy follow-up data. Analysis will be performed with Statistical Analysis Software version 9.4 (SAS Institute, Cary, NC, USA).

#### Qualitative analysis

Data from semi-structured interviews will be recorded, transcribed, anonymized and analyzed with QDA Miner software version 5 (Provalis Research, Montreal, QC, Canada). Content analysis and integration of quantitative inferences will be conducted based on an adaptation of the Interactive Quality Health Education Outcomes Model [56]. Administrative data will be treated in a descriptive manner in order to establish the general and comparative profile of users. Quality and confidentiality of data will be rigorously ensured by the use of consolidated criteria and validated qualitative methods [62, 63].

#### Multiple case study analysis

Case studies will consist of perinatal information networks of the two participating health and social services centers in which local community perinatal information networks will be embedded. For each study case, matrices allowing the evaluation of determinants in relation to networks success factors and collaborative actions presented in perinatal governmental programs [64–66] will be developed alongside a content analysis [67]. UCINET software version 6 [68] will be used to view and compare perinatal networks structure according to the analytical approach described by Scott et al. [69], as recommended by Provan et al. for the reinforcement of efficient collaboration networks [61]. The integration of different data sources will allow a cross-sectional validation of results.

### Collaboration with decision-makers

Decision-makers of the two participating health and social services centers have committed to facilitate the implementation of this project in their respective establishments. Based on the administrative data collected and usability of online prenatal education, they will standardize as much as possible their offer of group prenatal education and online prenatal education before the recruitment in order to optimize the results of the study. Responding to priorities in public health and clinical services organization, this engagement will facilitate a relevant follow-up of the impacts of group and online prenatal education. The study of the service delivery models for prenatal education and the associated regional networks providing these services will also promote collaboration between the political decision-makers of the Ministry of Health and Social Services of Québec, the National Public Health Institute of Québec, and the Public Health Agency of Canada who have agreed to actively participate in the interpretation of results and mobilization of knowledge strategies.

## Discussion

### Knowledge translation strategies

An advisory committee (composed of all authors, health professionals and managers as expert knowledge users) will support the development and operationalization of the study, notably for data collection follow-up and knowledge translation. A monitoring committee (composed of all authors, decision-makers, parent representatives and policy makers as expert knowledge users) will be responsible for sustained knowledge mobilization throughout all the study in order to support organizational and political decisions related to perinatal education services. This knowledge mobilization approach in its process, reflections, tools and results can be shared with the involved actors in order to disseminate the best practices in organizational terms for the users, the organizations, and partners of perinatal services networks. The use of brief reports, narrated slides and a website intended for the users, decision-makers, and partners will make up the principal knowledge transfer strategies and results dissemination.

### Expected outcomes

This study will be one of the first to consider the impacts of online prenatal education on different health determinants and perinatal health status in a Canadian context. This will allow for important knowledge acquisition regarding the impact of online prenatal education as a new technological service delivery model compared to an absence of group prenatal education in some health and social services centers settings. Indicators related to the complementarity of group and online prenatal education and those available in a community setting will refine our understanding of regional perinatal services networks. As studies involving future fathers or partners are uncommon, although their involvement in perinatal period is strongly recommended [70], results will also indicate how group and online prenatal education can contribute to their well-being and that of their family. This project also has the potential to improve harmonization of group prenatal education and the user-friendliness of online prenatal education. This could potentially improve nurses’ professional practices, as well as those of other health professionals and community stakeholders involved in perinatal education. The partnership approach will assist in the development of a measurement culture and support decision-making regarding service organization and delivery models of prenatal education in Québec as well as other Canadian provinces where online prenatal education are provided, in order to optimize perinatal health services.
